# Supplementary figures and images for: Long-term farming and cropping systems with contrasting nitrogen forms and input diversity influence soil prokaryotic diversity in the central highlands of Kenya
Source: PLoS One. 2026 Mar 16;21(3):e0344418. doi: 10.1371/journal.pone.0344418 (PMC12991241; doi:10.1371/journal.pone.0344418)

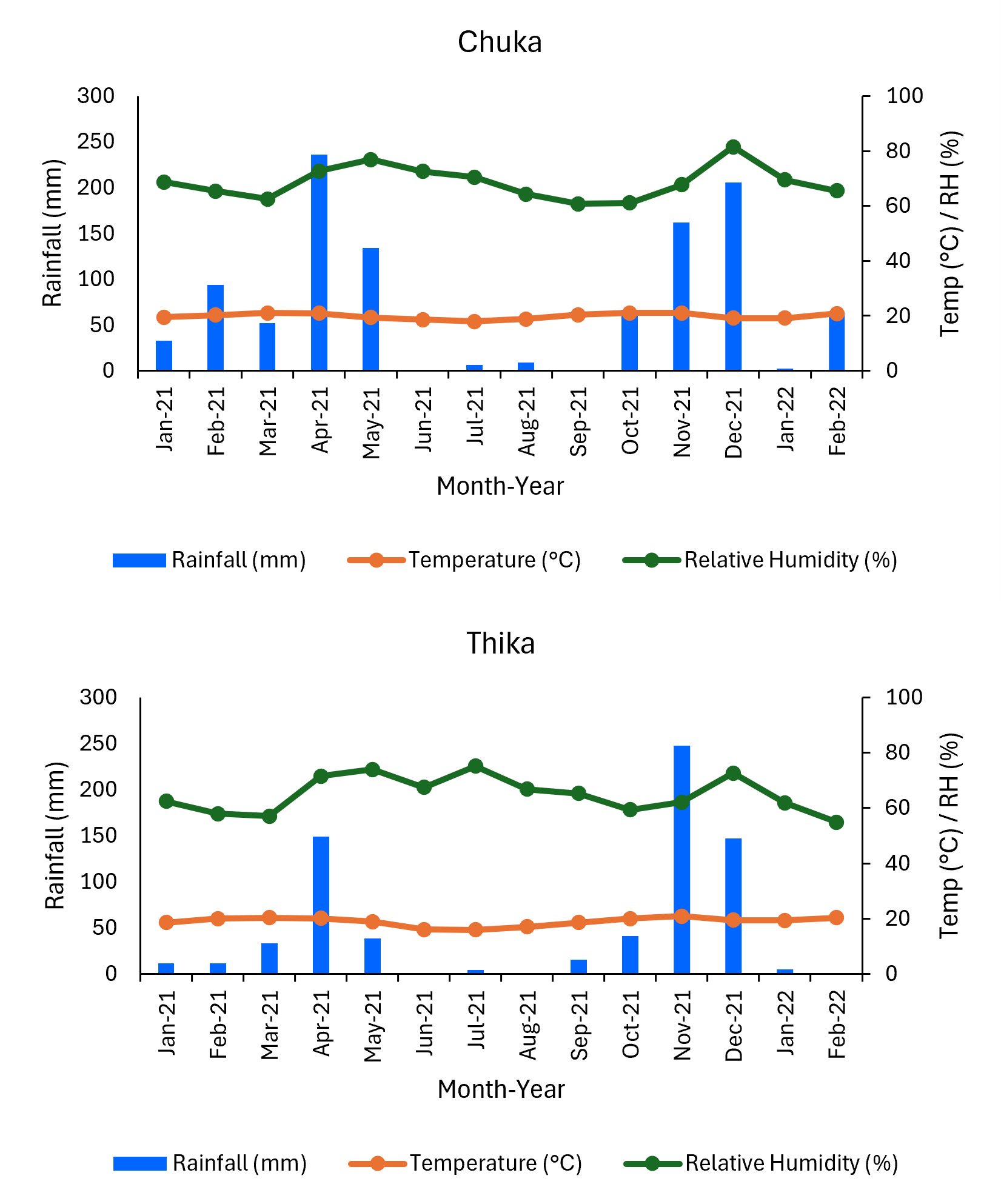

Supplement: S1 Fig — Monthly rainfall (mm, bars), mean temperature (°C, brown solid line), and relative humidity (% RH, green solid line) recorded during the study period at (A) Chuka and (B) Thika sites. (TIF) [file pone.0344418.s001.tif]
