# Supplementary material for: Long-term farming and cropping systems with contrasting nitrogen forms and input diversity influence soil prokaryotic diversity in the central highlands of Kenya
Source: PLoS One. 2026 Mar 16;21(3):e0344418. doi: 10.1371/journal.pone.0344418 (PMC12991241; doi:10.1371/journal.pone.0344418)
Supplement: S1 Table — Table presents soil chemical properties measured at the Chuka and Thika sites under contrasting farming systems and cropping arrangements after long-term management. Tables A and B show the effects of crop developmental stages on soil pH, total nitrogen, nitrate-N, ammonium-N, Olsen phosphorus, and total phosphorus at Chuka and Thika, respectively. Tables C and D summarize soil chemical characteristics across organic and conventional farming systems within different cropping systems at Chuka and Thika. Tables E and F present the interaction effects between crop growth stage and farming system on soil chemical properties at Chuka and Thika, respectively. Values are means of replicate soil samples. Different letters within a column indicate significant differences among treatments where applicable. Significance levels are indicated as p ≤ 0.05 (*), p ≤ 0.01 (**), p ≤ 0.001 (***), and ns denotes not significant. (DOCX) [file pone.0344418.s003.docx]

**S1 Table A-F.** Soil chemical characteristics across crop growth stages, farming systems, and sites in the SysCom Kenya long-term trial.

**Table (A).** Effect of crop developmental stages on soil chemical characteristics in the long-term farming systems comparison trials at Chuka.

| **Crop** | **Crop developmental stage** | **Soil pH** | **Total Nitrogen (%)** | **Nitrate N (mg kg)** | **Ammonium (mg kg)** | **Olsen P (mg kg)** | **Total P (mg kg)** |
| --- | --- | --- | --- | --- | --- | --- | --- |
| Babycorn desmodium | Vegetative | 5.66 | 0.22 | 5.84^a^ | 3.13^b^ | 71.05 | 1232.00 |
|  | Tussling and silking | 6.07 | 0.21 | 4.37^a^ | 3.70^b^ | 48.73 | 1307.50 |
|  | Grain formation | 6.10 | 0.23 | 1.91^b^ | 3.43^b^ | 65.48 | 1473.75 |
|  | Maturity | 5.74 | 0.23 | 5.35^a^ | 5.61^a^ | 61.96 | 1240.88 |
|  | p value | ns | ns | *** | ** | ns | ns |
| Maize bean intercrop | Vegetative | 5.60^ab^ | 0.18 | 3.51^b^ | 8.21^ab^ | 52.24^a^ | 1062.50^a^ |
|  | Tussling and silking | 5.90^a^ | 0.17 | 4.24^b^ | 3.21^b^ | 23.26^b^ | 833.13^b^ |
|  | Grain formation | 5.76^ab^ | 0.18 | 8.33^ab^ | 4.30^ab^ | 33.29^b^ | 957.00^ab^ |
|  | Maturity | 5.17^b^ | 0.19 | 15.77^a^ | 10.78^a^ | 38.74^ab^ | 928.88^ab^ |
|  | p value | * | ns | ** | * | *** | * |
| Potato dolichos desmodium | Vegetative | 6.20 | 0.32^a^ | 12.05^a^ | 7.45^ab^ | 61.23 | 1327.50^b^ |
|  | Flowering | 6.33 | 0.23^b^ | 3.00^b^ | 4.00^b^ | 65.79 | 1267.38^b^ |
|  | Maturity | 6.12 | 0.27^b^ | 11.90^a^ | 8.87^a^ | 58.88 | 1787.50^a^ |
|  | p value | ns | *** | *** | * | ns | *** |
| Potato dolichos intercrop | Vegetative | 5.88 | 0.25^a^ | 4.62^b^ | 4.29 | 25.13 | 846.25^b^ |
|  | Flowering | 6.01 | 0.22^b^ | 2.64^c^ | 5.61 | 30.40 | 852.38^b^ |
|  | Maturity | 5.95 | 0.20^b^ | 6.75^a^ | 24.66 | 31.08 | 1175.25^a^ |
|  | p value | ns | *** | *** | ns | ns | ** |

**Table (B).** Effect of crop developmental stages on soil chemical characteristics in the long-term farming systems comparison trials at Thika.

| **Crop** | **Crop developmental stage** | **Soil pH** | **Total Nitrogen (%)** | **Nitrate N (mg kg)** | **Ammonium (mg kg)** | **Olsen P (mg kg)** | **Total P (mg kg)** |
| --- | --- | --- | --- | --- | --- | --- | --- |
| Babycorn desmodium | Vegetative | 6.48 | 0.16^b^ | 3.39^c^ | 5.27^ab^ | 87.02 | 859.30^a^ |
|  | Tussling and silking | 6.61 | 0.15^b^ | 6.31^b^ | 4.76^b^ | 53.85 | 617.90^b^ |
|  | Grain formation | 6.71 | 0.19^a^ | 4.53^bc^ | 8.28^ab^ | 68.94 | 845.70^a^ |
|  | Maturity | 6.41 | 0.19^a^ | 10.61^a^ | 8.45^a^ | 99.82 | 708.10^ab^ |
|  | p value | ns | *** | *** | * | ns | *** |
| Maize bean intercrop | Vegetative | 5.93 | 0.11^b^ | 1.45^c^ | 1.78^b^ | 42.44 | 323.90^a^ |
|  | Tussling and silking | 6.03 | 0.11^b^ | 6.06^bc^ | 4.51^b^ | 14.30 | 110.38^c^ |
|  | Grain formation | 6.08 | 0.14^a^ | 13.02^ab^ | 8.11^b^ | 41.56 | 273.60^b^ |
|  | Maturity | 5.49 | 0.14^a^ | 15.12^a^ | 18.27^a^ | 37.75 | 300.30^ab^ |
|  | p value | ns | *** | *** | *** | ns | *** |
| Potato dolichos desmodium | Vegetative | 6.62 | 0.19^a^ | 6.62^b^ | 4.85 | 49.32 | 486.30^b^ |
|  | Flowering | 6.48 | 0.16^b^ | 6.00^b^ | 9.15 | 50.55 | 510.20^b^ |
|  | Maturity | 6.44 | 0.21^a^ | 18.35^a^ | 14.08 | 53.59 | 691.80^a^ |
|  | p value | ns | *** | *** | ns | ns | ** |
| Potato dolichos intercrop | Vegetative | 6.12 | 0.16^a^ | 2.93^b^ | 5.13^b^ | 18.70 | 351.80 |
|  | Flowering | 5.88 | 0.13^b^ | 4.51^b^ | 8.75^ab^ | 20.52 | 266.90 |
|  | Maturity | 6.01 | 0.16^a^ | 9.51^a^ | 14.82^a^ | 19.03 | 230.00 |
|  | p value | ns | ** | *** | * | ns | ns |

**Table (C).** Soil chemical characteristics in varied cropping systems under organic and conventional farming systems in the long-term system comparison trials at Chuka.

| **Crop** | **Farming system** | **Soil pH** | **Total Nitrogen (%)** | **Nitrate N (mg kg)** | **Ammonium (mg kg)** | **Olsen P (mg kg)** | **Total P (mg kg)** |
| --- | --- | --- | --- | --- | --- | --- | --- |
| Babycorn desmodium | Conv-High | 5.16^b^ | 0.19^b^ | 3.88 | 3.60 | 85.84^a^ | 1381.06 |
|  | Org-High | 6.63^a^ | 0.25^a^ | 4.85 | 4.33 | 37.76^b^ | 1246.00 |
|  | p value | *** | *** | ns | ns | *** | ns |
| Maize bean intercrop | Conv-Low | 5.21^b^ | 0.17 | 12.43^a^ | 9.46^a^ | 39.83 | 924.81 |
|  | Org-Low | 6.01^a^ | 0.18 | 3.50^b^ | 3.79^b^ | 33.93 | 965.94 |
|  | p value | *** | ns | *** | ** | ns | ns |
| Potato dolichos desmodium | Conv-High | 5.51^b^ | 0.25 | 8.38 | 6.84 | 80.67^a^ | 1463.33 |
|  | Org-High | 6.93^a^ | 0.29 | 9.59 | 6.71 | 43.26^b^ | 1458.25 |
|  | p value | *** | ns | ns | ns | *** | ns |
| Potato dolichos intercrop | Conv-Low | 5.67^b^ | 0.23 | 4.30 | 17.95 | 28.23 | 920.00 |
|  | Org-Low | 6.22^a^ | 0.22 | 5.04 | 5.08 | 29.50 | 995.92 |
|  | p value | *** | ns | ns | ns | ns | ns |

**Table (D).** Soil chemical characteristics in varied cropping systems under organic and conventional farming systems in the long-term system comparison trials at Thika.

| **Crop** | **Farming system** | **Soil pH** | **Total Nitrogen (%)** | **Nitrate N (mg kg)** | **Ammonium (mg kg)** | **Olsen P (mg kg)** | **Total P (mg kg)** |
| --- | --- | --- | --- | --- | --- | --- | --- |
| Babycorn desmodium | Conv-High | 5.81^b^ | 0.16^b^ | 6.62 | 6.00 | 120.72^a^ | 705.25^b^ |
|  | Org-High | 7.29^a^ | 0.19^a^ | 5.80 | 7.38 | 34.10^b^ | 810.25^a^ |
|  | p value | *** | *** | ns | ns | *** | * |
| Maize bean intercrop | Conv-Low | 5.72^b^ | 0.13 | 10.00^a^ | 7.73 | 34.37 | 255.83 |
|  | Org-Low | 6.28^a^ | 0.13 | 4.53^b^ | 4.93 | 31.66 | 248.41 |
|  | p value | *** | ns | *** | ns | ns | ns |
| Potato dolichos desmodium | Conv-High | 5.83^b^ | 0.18 | 11.89 | 13.66^a^ | 76.98^a^ | 493.27^b^ |
|  | Org-High | 7.19^a^ | 0.19 | 8.75 | 5.06^b^ | 25.33^b^ | 632.27^a^ |
|  | p value | *** | ns | ns | * | *** | * |
| Potato dolichos intercrop | Conv-Low | 5.70^b^ | 0.15 | 6.31 | 13.02^a^ | 20.11 | 226.67 |
|  | Org-Low | 6.31^a^ | 0.15 | 4.98 | 6.11^b^ | 18.73 | 339.13 |
|  | p value | *** | ns | ns | * | ns | ns |

**Table (E).** Interaction effect of crop growth stages and farming systems on soil chemical characteristics at Chuka.

| **Crop** | **Crop growth stage** | **Farming system** | **Soil pH** | **Total Nitrogen (%)** | **Nitrate N (mg kg)** | **Ammonium (mg kg)** | **Olsen P (mg kg)** | **Total P (mg kg)** |
| --- | --- | --- | --- | --- | --- | --- | --- | --- |
| Babycorn desmodium | Vegetative | Conv-High | 4.99b | 0.21b | 6.83 | 3.43 | 98.33a | 1442.50 |
|  |  | Org-High | 6.34a | 0.24a | 4.85 | 2.83 | 43.78b | 1021.50 |
|  | p value |  | *** | *** | ns | ns | *** | ns |
|  | Tussling and silking | Conv-High | 5.39b | 0.18b | 3.75b | 3.86 | 69.78a | 1367.50 |
|  |  | Org-High | 6.75a | 0.25a | 5.00a | 3.55 | 27.68b | 1247.50 |
|  | p value |  | *** | *** | ** | ns | *** | ns |
|  | Grain formation | Conv-High | 5.36b | 0.19b | 1.24 | 1.83b | 90.18a | 1497.50 |
|  |  | Org-High | 6.84a | 0.27a | 2.57 | 5.04a | 40.78b | 1450.00 |
|  | p value |  | *** | ** | ns | ** | ** | ns |
|  | Maturity | Conv-High | 4.90b | 0.20b | 3.70 | 5.30 | 85.10a | 1216.75 |
|  |  | Org-High | 6.78a | 0.26a | 7.00 | 5.91 | 38.83b | 1265.00 |
|  | p value |  | *** | * | ns | ns | *** | ns |
| Crop growth stage | | | *** | ns | *** | *** | *** | * |
| Farming system | | | *** | *** | ns | ns | *** | * |
| Crop growth stage × Farming system | | | * | ns | * | ** | ns | ns |
| Maize bean intercrop | Vegetative | Conv-Low | 5.17b | 0.17 | 6.23a | 12.50 | 60.08 | 1067.75 |
|  |  | Org-Low | 6.03a | 0.19 | 0.79b | 3.93 | 44.40 | 1057.25 |
|  | p value |  | ** | ns | ** | ns | ns | ns |
|  | Tussling and silking | Conv-Low | 5.57 | 0.16 | 5.28 | 3.48 | 24.33 | 851.75 |
|  |  | Org-Low | 6.24 | 0.17 | 3.21 | 2.94 | 22.20 | 814.50 |
|  | p value |  | * | ns | ns | ns | ns | ns |
|  | Grain formation | Conv-Low | 5.40b | 0.18 | 13.32a | 6.56a | 33.50 | 889.75 |
|  |  | Org-Low | 6.12a | 0.19 | 3.34b | 2.04b | 33.08 | 1024.25 |
|  | p value |  | * | ns | ** | ** | ns | ns |
|  | Maturity | Conv-Low | 4.70b | 0.19 | 24.90a | 15.29 | 41.43 | 890.00 |
|  |  | Org-Low | 5.64a | 0.19 | 6.65b | 6.26 | 36.05 | 967.75 |
|  | p value |  | ** | ns | ** | ns | ns | ns |
| Crop growth stage | | | *** | ns | *** | ** | *** | *** |
| Farming system | | | *** | ns | *** | *** | ns | ns |
| Crop growth stage × Farming system | | | ns | ns | ** | ns | ns | ns |
| Potato dolichos desmodium | Vegetative | Conv-High | 5.47b | 0.36 | 9.89 | 9.84 | 76.63 | 1402.50 |
|  |  | Org-High | 6.93a | 0.29 | 14.20 | 5.06 | 45.83 | 1252.50 |
|  | p value |  | *** | ** | ns | ns | *** | ns |
|  | Flowering | Conv-High | 5.64b | 0.23 | 3.49 | 3.77 | 85.78a | 1225.00 |
|  |  | Org-High | 7.02a | 0.22 | 2.52 | 4.23 | 45.80b | 1309.75 |
|  | p value |  | *** | ns | ns | ns | *** | ns |
|  | Maturity | Conv-High | 5.42b | 0.24b | 11.77 | 11.69a | 79.60a | 1762.50 |
|  |  | Org-High | 6.83a | 0.30a | 12.04 | 6.06b | 38.15b | 1812.50 |
|  | p value |  | *** | *** | ns | * | * | ns |
| Crop growth stage | | | * | *** | *** | * | ns | *** |
| Farming system | | | *** | ** | ns | ns | *** | ns |
| Crop growth stage × Farming system | | | ns | * | ns | ** | ns | ns |
| Potato dolichos intercrop | Vegetative | Conv-Low | 5.66 | 0.25 | 4.29 | 3.01 | 25.88 | 838.50 |
|  |  | Org-Low | 6.11 | 0.26 | 4.95 | 5.56 | 24.38 | 854.00 |
|  | p value |  | ns | ns | ns | ns | ns | ns |
|  | Flowering | Conv-Low | 5.69b | 0.24 | 3.36 | 8.25a | 28.28 | 791.00 |
|  |  | Org-Low | 6.33a | 0.20 | 1.92 | 2.98b | 32.53 | 913.75 |
|  | p value |  | * | ns | ns | * | ns | ns |
|  | Maturity | Conv-Low | 5.67b | 0.20 | 5.24b | 42.60 | 30.55 | 1130.50 |
|  |  | Org-Low | 6.24a | 0.19 | 8.25a | 6.71 | 31.60 | 1220.00 |
|  | p value |  | * | ns | * | ns | ns | ns |
| Crop growth stage | | | ns | *** | *** | ns | ns | *** |
| Farming system | | | *** | ns | ns | ns | ns | ns |
| Crop growth stage × Farming system | | | ns | * | ** | ns | ns | ns |

**Table (F).** Interaction effect of crop growth stages and farming systems on soil chemical characteristics at Thika.

| **Crop** | **Crop growth stage** | **Farming system** | **Soil pH** | **Total Nitrogen (%)** | **Nitrate N (mg kg)** | **Ammonium (mg kg)** | **Olsen P (mg kg)** | **Total P (mg kg)** |
| --- | --- | --- | --- | --- | --- | --- | --- | --- |
| Babycorn desmodium | Vegetative | Conv-High | 5.80^b^ | 0.15^b^ | 4.91^a^ | 5.44 | 124.40^a^ | 741.40^b^ |
|  |  | Org-High | 7.16^a^ | 0.17^a^ | 1.86^b^ | 5.09 | 49.64^b^ | 977.20^a^ |
|  | p value |  | *** | * | ** | ns | *** | * |
|  | Tussling and silking | Conv-High | 5.91^b^ | 0.14^b^ | 6.00 | 4.35 | 84.92^a^ | 637.40 |
|  |  | Org-High | 7.30^a^ | 0.16^a^ | 6.61 | 5.17 | 22.78^b^ | 598.40 |
|  | p value |  | *** | * | ns | ns | *** | ns |
|  | Grain formation | Conv-High | 5.88^b^ | 0.17^b^ | 4.64 | 5.40^b^ | 104.74^a^ | 766.00^b^ |
|  |  | Org-High | 7.54^a^ | 0.22^a^ | 4.42 | 11.16^a^ | 33.14^b^ | 925.40^a^ |
|  | p value |  | *** | *** | ns | ** | *** | ** |
|  | Maturity | Conv-High | 5.66^b^ | 0.17^b^ | 10.91 | 8.82 | 168.80^a^ | 676.20 |
|  |  | Org-High | 7.16^a^ | 0.21^a^ | 10.31 | 8.08 | 30.84^b^ | 740.00 |
|  | p value |  | *** | ** | ns | ns | *** | ns |
| Crop growth stage | | | ** | *** | *** | ** | *** | *** |
| Farming system | | | *** | *** | ns | ns | *** | ** |
| Crop growth stage × Farming system | | | ns | ns | * | * | *** | ns |
| Maize bean intercrop | Vegetative | Conv-Low | 5.61^b^ | 0.11 | 2.28 | 2.29 | 63.00^a^ | 341.20 |
|  |  | Org-Low | 6.26^a^ | 0.12 | 0.62 | 1.27 | 21.88^b^ | 306.60 |
|  | p value |  | ** | ns | ns | ns | *** | ns |
|  | Tussling and silking | Conv-Low | 5.62^b^ | 0.11 | 7.72^a^ | 4.32 | 12.89 | 105.72 |
|  |  | Org-Low | 6.44^a^ | 0.11 | 4.40^b^ | 4.70 | 15.70 | 115.04 |
|  | p value |  | * | ns | ** | ns | ns | ns |
|  | Grain formation | Conv-Low | 5.70^b^ | 0.14 | 21.48^a^ | 13.40^a^ | 27.84 | 275.80 |
|  |  | Org-Low | 6.46^a^ | 0.14 | 4.55^b^ | 2.83^b^ | 55.28 | 271.40 |
|  | p value |  | ** | ns | *** | *** | ns | ns |
|  | Maturity | Conv-Low | 5.02^b^ | 0.14 | 21.70^a^ | 25.62^a^ | 41.74 | 300.00 |
|  |  | Org-Low | 5.96^a^ | 0.14 | 8.54^b^ | 10.92^b^ | 33.76 | 300.60 |
|  | p value |  | ** | ns | ** | * | ns | ns |
| Crop growth stage | | | *** | *** | *** | *** | ns | *** |
| Farming system | | | *** | ns | *** | *** | ns | ns |
| Crop growth stage × Farming system | | | ns | ns | *** | ** | ns | ns |
| Potato dolichos desmodium | Vegetative | Conv-High | 6.05^b^ | 0.18 | 5.95 | 5.29 | 73.20^a^ | 482.60 |
|  |  | Org-High | 7.19^a^ | 0.20 | 7.29 | 4.41 | 25.44^b^ | 490.00 |
|  | p value |  | *** | ns | ns | ns | *** | ns |
|  | Flowering | Conv-High | 5.76^b^ | 0.16 | 8.42^a^ | 15.29 | 78.12^a^ | 439.20^b^ |
|  |  | Org-High | 7.20^a^ | 0.16 | 3.57^b^ | 3.01 | 22.98^b^ | 581.20^a^ |
|  | p value |  | *** | ns | * | ns | *** | * |
|  | Maturity | Conv-High | 5.69^b^ | 0.20 | 21.29 | 20.41 | 79.62^a^ | 558.00^b^ |
|  |  | Org-High | 7.19^a^ | 0.22 | 15.40 | 7.76 | 27.56^b^ | 825.60^a^ |
|  | p value |  | *** | ns | ns | ns | *** | *** |
| Crop growth stage | | | ns | *** | *** | ns | ns | *** |
| Farming system | | | *** | ns | ns | * | *** | *** |
| Crop growth stage × Farming system | | | ns | ns | ns | ns | ns | * |
| Potato dolichos intercrop | Vegetative | Conv-Low | 5.85 | 0.16 | 3.03 | 5.59 | 19.42 | 205.80 |
|  |  | Org-Low | 6.39 | 0.16 | 2.82 | 4.66 | 17.98 | 497.80 |
|  | p value |  | ns | ns | ns | ns | ns | ns |
|  | Flowering | Conv-Low | 5.53 | 0.13 | 6.64 | 13.78^a^ | 22.74 | 254.40 |
|  |  | Org-Low | 6.23 | 0.13 | 2.39 | 3.73^b^ | 18.30 | 279.40 |
|  | p value |  | ns | ns | ns | ** | ns | ns |
|  | Maturity | Conv-Low | 5.70^b^ | 0.15 | 9.27 | 19.69 | 18.16 | 219.80 |
|  |  | Org-Low | 6.31^a^ | 0.16 | 9.74 | 9.95 | 19.90 | 240.20 |
|  | p value |  | * | ns | ns | ns | ns | ns |
| Crop growth stage | | | ns | *** | *** | ** | ns | ns |
| Farming system | | | *** | ns | ns | ** | ns | ns |
| Crop growth stage × Farming system | | | ns | ns | ns | ns | ns | ns |
