# Supplementary material for: Long-term farming and cropping systems with contrasting nitrogen forms and input diversity influence soil prokaryotic diversity in the central highlands of Kenya
Source: PLoS One. 2026 Mar 16;21(3):e0344418. doi: 10.1371/journal.pone.0344418 (PMC12991241; doi:10.1371/journal.pone.0344418)
Supplement: Attachment — Submitted filename: Report on PONE-D-25-53620.docx [file pone.0344418.s004.docx]

Comments on "Nitrogen forms and cropping systems influence soil prokaryotic diversity within organic and conventional farming systems in the central highlands of Kenya” (PONE-D-25-53620)

The authors examined how different farming systems and nitrogen management strategies affect soil prokaryotic diversity in Kenya and found significant variation in microbial diversity and composition based on farming systems, input intensity, and crop growth stages. Though they found that diversity peaked consistently at crop maturity, they did not discuss the reasons for this peak at grain formation and maturity. The canonical correspondence analysis identified ammonium-N, available phosphorus, and pH as strong correlates of microbial community structure, especially at Thika. What were the soil organic carbon contents of these soils? Several genera, including *Rubinisphaera*, *Methanomassiliicoccus*, and *Microvirga*, were enriched in organic systems, indicating microbial specialization at different growth stages. But the authors made no efforts to analyse the functional contributions of soil prokaryotes. Their findings support and provide additional evidence that organic high-input systems can foster beneficial microbial communities and emphasize the importance of understanding microbial ecology to improve agricultural productivity in sub-Saharan Africa, where soil fertility is a significant challenge.

The authors relied more on alpha- and beta-diversity measures. Alpha diversity is an ambiguous concept that encompasses several complementary aspects. The assumptions underlying alpha diversity metrics are not always directly meaningful or provide a clear interpretation of results. The choice of the diversity metrics influences the subsequent statistical testing. The authors acknowledged the limitations of their study in Lines 469-471 (*Because sequencing was conducted on pooled samples, replication at the plot level was not possible. This constrains statistical resolution and limits our ability to test treatment effects with full confidence*). Discussion needs to include the limitations of the diversity metrics analysed and of the 16S rRNA surveys (amplicon sequencing).

Line 182: universal primers, instead of universal bacterial primers. (*Methanomassiliicoccus* – an archaeal genus, not a bacterial genus).

Table 1: Simpson and Inverse Simpson? Check the entries. The theoretical range of values of the Simpson index (D) is between 0 and 1. The range of Shannon index values for soil bacterial ASVs varies significantly across soil environments, but typical values in ecological studies are between 1.5 and 3.5.

Line 420: What were the unique insights?

I recommend the present manuscript after incorporating the suggestions for publication in *PLOS ONE.*
