# Supplementary material for: Long-term farming and cropping systems with contrasting nitrogen forms and input diversity influence soil prokaryotic diversity in the central highlands of Kenya
Source: PLoS One. 2026 Mar 16;21(3):e0344418. doi: 10.1371/journal.pone.0344418 (PMC12991241; doi:10.1371/journal.pone.0344418)
Supplement: Attachment — Submitted filename: Response to Reviewers .docx [file pone.0344418.s005.docx]

**Response to Reviewers**

**Manuscript ID:** PONE-D-25-53620

**Title:** *Long-term farming and cropping systems with contrasting nitrogen forms and input intensities influence soil prokaryotic diversity in the Central Highlands of Kenya*

We sincerely thank the Academic Editor and the reviewers for their thorough, insightful, and constructive comments. All concerns raised in the reviewer reports and decision letter have been carefully addressed. The manuscript has been revised to improve clarity, rigor, interpretation, and alignment with the long-term experimental context. Below, we provide a detailed point-by-point response indicating how each comment has been addressed in the revised manuscript.

**Reviewer #1**

**General comment:** *The presentation and discussion of results are too general and superficial.*

**Response:** We appreciate this feedback and have strengthened the manuscript, particularly the Introduction, Results, Discussion and Conclusion (lines 53-111; 277-479; 480-573; 574-597). We expanded the interpretation of results, explicitly reconciled findings with earlier SysCom Kenya studies, clarified methodological limitations, refined conclusions, and ensured that interpretations are proportionate to the data. The revised Discussion now provides deeper ecological context while maintaining appropriate caution.

**Major Comment 1:** *Need to set the stage by discussing findings from Karanja et al. (2020) and explain why results differ.*

**Response:** We fully agree and now explicitly compare our findings with Karanja et al. (2020) in the Introduction and Discussion (lines 74-80; 486-499). We discuss how differences in temporal scale (early vs. 15 years of cumulative management), system maturity, organic input diversity, crop rotations, sampling periods, and sequencing pipelines likely explain the contrasting diversity patterns observed between studies.

**Major Comment 2:** *Need for hypotheses and concern that the study is descriptive, with no statistical testing of diversity indices.*

**Response:** We acknowledge this concern and we have added a hypothesis statement (lines 93-100). We have also clarified the scope and limitations of the study. Due to pooling of soil samples for sequencing, formal inferential statistical testing of microbial diversity indices was not possible. This limitation is now clearly stated in the Abstract, Methods and Discussion (lines 47-49; 184-187; 559-568), and microbial results are explicitly interpreted as system-level patterns. Statistical analyses were retained for soil chemical variables measured at the replicate level. The Discussion now clearly distinguishes descriptive microbial patterns from statistically tested abiotic relationships.

**Major Comment 3:** *Need means and standard deviations of soil variables; Zenodo links did not contain soil chemistry data.*

**Response:** We thank the reviewer for identifying this issue. The soil chemical data initially cited to be in the Zenodo repository, has been provided as supporting information for all systems, sites, and sampling periods (S1 Table A-F). The Data Availability statement has been revised accordingly.

**Major Comment 4:** *Emphasize organic input diversity rather than nitrogen form; all systems received manure.*

**Response:** We agree and have revised the manuscript to emphasize organic input diversity and input intensity as primary explanatory factors rather than nitrogen form alone. This clarification is reflected in the Discussion (Lines 507-512) subsection as well as in the Abstract (lines 45-47) and Title (lines 1-3). We also refer readers to nutrient input summaries provided in Karanja et al. (2020) for long-term context.

**Major Comment 5:** *Check reference numbering and suitability of citations.*

**Response:** All references have been carefully reviewed and corrected to ensure that citations accurately support the statements made in the text.

**Major Comment 6:** *Figure 8 is wrong (fungal taxa); LEfSe analysis not meaningful.*

**Response:** We fully agree. The incorrect fungal LEfSe figure has been removed and replaced with the correct prokaryotic LEfSe figure (lines 471). The Results (lines 457-470) and Discussion text (Lines 530-536) have been revised to interpret LEfSe outputs cautiously as descriptive indicators of taxonomic differentiation, not functional dominance, or statistically tested effects.

**Major Comment 7:** *Sequences not deposited in NCBI.*

**Response:** Sequence data had been deposited but not released to the public, but now the data has been released; by using the provided link, one can now access the data deposited in the NCBI Sequence Read Archive. The BioProject accession number is provided in the revised Data Availability section (lines 614-619).

**Reviewer #1 - Other Comments**

**Comment 1 (Methods: Tillage practices):** *Please explain the type of tillage used locally.*

**Response:** We clarified that tillage followed local smallholder practices, consisting of shallow conventional tillage using hand hoes without deep inversion tillage. This information has been added to the Methods section (lines 151-153).

**Comment 2 (Methods: DNA extraction efficiency in Nitisols):** *DNA extraction may be inefficient in clay-rich soils; provide DNA yield information if available.*

**Response:** We acknowledge this limitation. Mean DNA yields per gram of dry soil were not consistently archived and could not be reported. However, to improve DNA yield during extraction from the nitisols soil which are known to have high clay content, mechanical disruption was applied which involved use of bead beating tubes to increase the recovery of DNA in the soil (lines 206-208).

**Comment 3 (Results: Figure 1 placement):** *Figure 1 could be moved to supplemental files.*

**Response:** We agree and have moved the climate figure to the Supporting/Supplementary Information (S1 Fig) to improve focus in the main Results section.

**Comment 4 (Results: CCA variables):** *Were community compositions tested only against pH, ammonium, and available P?*

**Response:** All measured soil chemical variables were initially included in ordination analyses. Following multicollinearity screening and variable selection, only pH, ammonium-N, and available phosphorus were retained in the final CCA models. This process is now explicitly described (lines 320-330; 338-347), and full soil chemistry data are provided in the Supporting information (S1 Table A-F).

**Comment 5 (Results: Figure 2 legend):** *The legend should explain color coding.*

**Response:** Figure legends have been revised to clearly explain how colors and symbols represent farming systems and crop growth stages.

**Comment 6 (Results: Figure 3 clarity):** *Points should have both colors and shapes; interpretation needs clarification.*

**Response:** The Results text has been expanded to clarify that clustering reflects stronger system-level effects relative to crop growth stage effects and the arrows represent the direction and strength of correlations with individual soil variables (lines 349-354).

**Comment 7 (Results: Dominance wording):** *It would be more accurate to say Proteobacteria and Actinobacteria were dominant.*

**Response:** We agree and have revised the wording accordingly in the Results section (lines 357-358).

**Comment 8 (Results: Low-abundance phyla):** *Chloroflexi and Firmicutes are hard to see; is this worth mentioning?*

**Response:** We reduced emphasis on low-abundance phyla and now mention them only where consistent patterns were observed across systems or stages.

**Comment 9 (Results/Discussion: Input diversity emphasis):** *Emphasize organic input diversity rather than nitrogen source.*

**Response:** We revised Title (lines 1-3), Abstract (lines 45-47), and Discussion (lines 507-518) sections to emphasize organic input diversity and management intensity rather than nitrogen form alone.

**Comment 10 (Results: Phenology effects):** *Differences do not look significant; system effects seem stronger.*

**Response:** We revised the Results text to avoid implying statistical significance and clarified that system-level effects were more consistent than phenological effects (lines 320-456).

**Comment 11 (Discussion depth):** *Discussion is too generalized.*

**Response:** The Discussion has been fully revised and expanded to provide deeper, system-specific interpretation and reconciliation with earlier SysCom Kenya findings (lines 480-573).

**Comment 12 (Figure 8 inconsistency):** *Figure 8 shows fungal taxa.*

**Response:** The incorrect fungal figure was removed and replaced with the correct prokaryotic LEfSe figure (lines 471). The Results (lines 457-470) and Discussion (Lines 530-536) text have been revised to interpret LEfSe outputs cautiously as descriptive indicators of taxonomic differentiation, not functional dominance or statistically tested effects.

**Comment 13 (Phenology centrality):** *Not sure crop phenology played a central role.*

**Response:** We revised the Discussion (lines 500-506; 519-536) and Conclusion (lines 581-585) to clarify that crop growth stage is a secondary driver relative to long-term system management.

**Reviewer #2**

We thank Reviewer #2 for the positive evaluation and constructive suggestions.

**Comment 1:** *Reasons for diversity peaking at maturity not discussed.*

**Response:** We expanded the Discussion (lines 519-523) to explain increases toward later crop stages in terms of rhizodeposition, root turnover, and residue inputs, while emphasizing variability and secondary importance of phenology.

**Comment 2:** *What were soil organic carbon contents?*

**Response:** Soil organic carbon was not measured. This limitation is now explicitly acknowledged, and its implications discussed, with recommendations for future work (lines 545-548).

**Comment 3:** *No functional analysis of prokaryotes.*

**Response:** We clarified that 16S rRNA sequencing provides taxonomic resolution only and that functional interpretations are inferential. Future functional approaches are outlined (lines 559-564).

**Comment 4:** *Limitations of alpha diversity metrics and amplicon sequencing.*

**Response:** We expanded the Discussion (lines 554-568) to address conceptual and methodological limitations of alpha diversity metrics and amplicon sequencing approaches.

**Comment 5:** *Primer terminology incorrect.*

**Response:** Terminology has been corrected to “universal prokaryotic 16S rRNA gene primers” throughout the manuscript (lines 210-212).

**Comment 6:** *Table 1 diversity indices inconsistent.*

**Response:** We acknowledge that incorrect values were previously reported in the table. The values for high-quality sequences, observed ASVs, Shannon diversity, Simpson index, and Fisher’s alpha for all treatments and sites have now been carefully checked and corrected in the revised Table 1 (314-319).

**Comment 7:** *Clarify unique insights.*

**Response:** We revised the Discussion (lines 569-573) and Conclusion (lines 575-597) to clearly articulate the unique contribution of this 15-year long-term study from sub-Saharan Africa.

**Editorial / Report Comments**

We confirm that all comments provided in the reviewer reports and decision letter have been fully addressed. Overlapping concerns were consolidated for clarity, while ensuring complete traceability between comments and revisions.

**Response to Journal Requirements**

We thank the editorial office for outlining the additional requirements for revision. We have carefully addressed each point as detailed below.

**Requirement 1:** *PLOS ONE style and file naming*

**Response:** We confirm that the revised manuscript has been formatted to fully comply with *PLOS ONE* style requirements, including manuscript structure, headings, references, figures, tables, and file naming conventions. The formatting has been checked against the official PLOS ONE templates for the main body and title/authors/affiliations.

**Requirement 2:** *Amended Funding Statement and declaration of all support*

**Response:** Thank you for this clarification. We have amended the Funding Statement to explicitly declare all funding and sources of support received during the study, in accordance with the PLOS ONE Guide for Authors.

Amended Funding Statement (for update in the online submission form): *The authors gratefully acknowledge the financial support for this research by the following organizations and agencies: Biovision Foundation (Grant No: 1040), Coop Sustainability Fund (Grant No: 1040), Liechtenstein Development Service (LED) (Grant No: 1040), and the Swiss Agency for Development and Cooperation (SDC) (Grant No: 1040); the Swedish International Development Cooperation Agency (Sida); the Swiss Agency for Development and Cooperation (SDC); the Australian Centre for International Agricultural Research (ACIAR); the Government of Norway; the German Federal Ministry for Economic Cooperation and Development (BMZ); and the Government of the Republic of Kenya. There was no additional external funding received for this study. The views expressed herein do not necessarily reflect the official opinion of the donors.*

We kindly request that this amended Funding Statement be updated in the online submission form on our behalf.

**Requirement 3:** *Removal of funding information from the manuscript*

**Response:** We have removed all funding-related text from the Acknowledgments section and any other sections of the manuscript. Funding information is now reported only through the Funding Statement for inclusion in the online submission form, in line with PLOS ONE policy.

**Requirement 4:** *Inclusion of author Edward Karanja in submission data*

**Response:** We confirm that the manuscript submission data have been amended via *Edit Submission* to include Edward Nderitu Karanja as an author.

**Requirement 5:** *Authorship list in manuscript file*

**Response:** We have updated the authorship list in the manuscript file to include the full name Edward Nderitu Karanja, ensuring consistency between the manuscript and submission metadata.

**Requirement 6:** *Ethics statement placement*

**Response:** We confirm that the ethics statement previously appearing in Declaration section has been removed.

**Requirement 7:** *Upload of revised figures*

**Response:** We have uploaded new, high-resolution versions of Figures 1, 2, 3, 5, 6, 7, Supplementary S1 Fig, and S2 Fig ensuring improved clarity, legibility, and compliance with PLOS ONE figure preparation guidelines.

We trust that these revisions fully address the journal’s requirements. We appreciate the editorial guidance and thank you for your assistance in updating the online submission information on our behalf.

**Final statement**

We thank the Academic Editor and reviewers for their constructive feedback. We believe the revisions have strengthened the manuscript and clarified its contributions and limitations. We hope the revised version satisfactorily addresses all concerns and remain happy to make any further adjustments if required.
